# Supplementary material for: Identification of photoperiod-induced specific miRNAs in the adrenal glands of Sunite sheep (Ovis aries)
Source: Front Vet Sci. 2022 Jul 22;9:888207. doi: 10.3389/fvets.2022.888207 (PMC9354845; doi:10.3389/fvets.2022.888207)
Supplement: Supplementary file 1 [file Data_Sheet_1.ZIP › Supplementary file/miRDeep2 file.zip!/miRDeep2 file/Novel_Structure/SP42A2/Novel_493.pdf]

Provisional ID	:	NC_019478.2_36073
Score total	:	2.2
Score for star read(s)	:	-1.3
Score for read counts	:	0
Score for mfe	:	1.9
Score for randfold	:	1.6
Score for cons. seed	:	
Total read count	:	97
Mature read count	:	97
Loop read count	:	0
Star read count	:	0

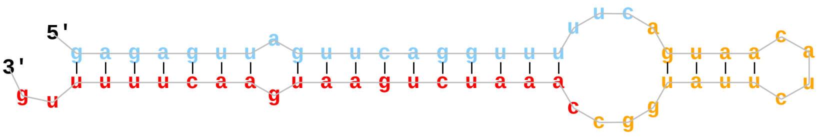

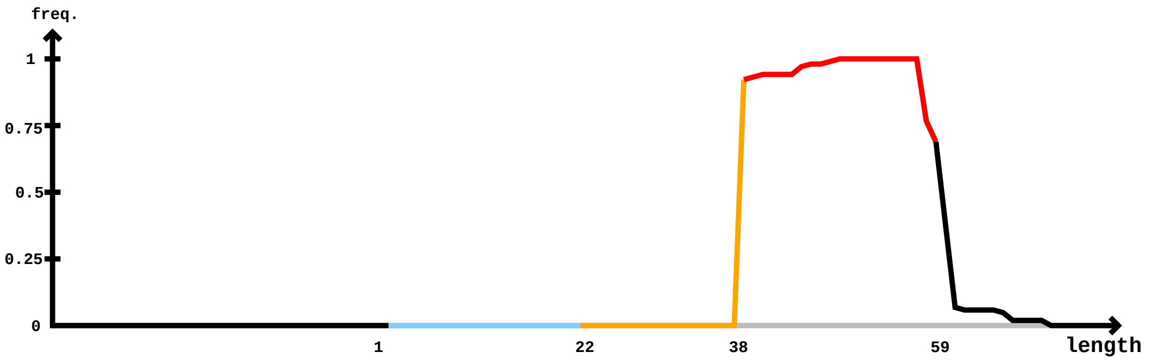

Star   Mature

5'- gagaagacggaucugagcgcuacugggugg ccagagaguuaguucagguuuuucaguaacaucuuauggccaaaucugaaugaacuuuuuggccaacucaguacacaga -3'	exp		
...........((((...(.((((((((((((((((((((.((((((((((....((((....))))....)))))))))).)))))))))))).))))))))).))))	reads	mm	sample
....caaaucugaaugaacuuuu....................	1	0	seq
....Aaaaucugaaugaacuuuu....................	23	1	seq
....caaaucugaaugaacuuuuu...................	7	0	seq
....Aaaaucugaaugaacuuuuug..................	57	1	seq
....caaaucugaaugaacuuuuugg.................	7	0	seq
....aaaucugaaugaacuuuuuggc................	1	0	seq
....aaucugaaugaacuuuuu...................	1	0	seq
....uAaaugaacuuuuuggccaac............	1	1	seq
....ugaGugaacuuuuuggccaacu...........	2	1	seq
....gaaCgaacuuuuuggccaacu...........	1	1	seq
....augaacuuuuuggccaacCcagu.......	1	1	seq
....ugaacuuuuuggccaacucagu.......	1	0	seq
